# Clinical utility of *PKD2* mutation testing in a polycystic kidney disease cohort attending a specialist nephrology out-patient clinic

**DOI:** 10.1186/1471-2369-13-79

**Published:** 2012-08-03

**Authors:** Caroline Robinson, Thomas F Hiemstra, Deborah Spencer, Sarah Waller, Laura Daboo, Fiona E Karet Frankl, Richard N Sandford

**Affiliations:** 1 Academic Department of Medical Genetics, University of Cambridge School of Clinical Medicine, Cambridge, CB2 0SP, UK; 2 Cambridge Institute for Medical Research, Cambridge Institute for Medical Research, Cambridge, CB2 0XY, UK; 3 East Anglian Medical Genetics Service, Addenbrooke’s Hospital, Cambridge, CB2 0QQ, UK

## Abstract

**Background:**

ADPKD affects approximately 1:1000 of the worldwide population. It is caused by mutations in two genes, *PKD1* and *PKD2*. Although allelic variation has some influence on disease severity, genic effects are strong, with *PKD2* mutations predicting later onset of ESRF by up to 20 years. We therefore screened a cohort of ADPKD patients attending a nephrology out-patient clinic for *PKD2* mutations, to identify factors that can be used to offer targeted gene testing and to provide patients with improved prognostic information.

**Methods:**

142 consecutive individuals presenting to a hospital nephrology out-patient service with a diagnosis of ADPKD and CKD stage 4 or less were screened for mutations in *PKD2*, following clinical evaluation and provision of a detailed family history (FH).

**Results:**

*PKD2* mutations were identified in one fifth of cases. 12% of non-*PKD2* patients progressed to ESRF during this study whilst none with a *PKD2* mutation did (median 38.5 months of follow-up, range 16–88 months, *p* < 0.03). A significant difference was found in age at ESRF of affected family members (non-*PKD2* vs. *PKD2*, 54 yrs vs. 65 yrs; *p* < 0.0001). No *PKD2* mutations were identified in patients with a FH of ESRF occurring before age 50 yrs, whereas a *PKD2* mutation was predicted by a positive FH without ESRF.

**Conclusions:**

*PKD2* testing has a clinically significant detection rate in the pre-ESRF population. It did not accurately distinguish those individuals with milder renal disease defined by stage of CKD but did identify a group less likely to progress to ESRF. When used with detailed FH, it offers useful prognostic information for individuals and their families. It can therefore be offered to all but those whose relatives have developed ESRF before age 50.

## Background

ADPKD is an important monogenic cause of renal disease worldwide, affecting more than 1:1000 of the population. It accounted for 6.7% of incident cases in the UK in 2009 requiring RRT and 9.6% of the prevalent cases (UK Renal Registry, http://www.renalreg.com). The development of ESRF typically occurs in later life, although there is considerable inter- and intra-familial variability [1]. Factors that have some minor predictive value for disease severity and earlier onset of ESRF include male sex, early age of diagnosis, early onset hypertension, and macroscopic haematuria [2]. Therefore it is difficult to provide accurate prognostic information to affected individuals and their at-risk family members. Although clinical trials are in progress, there are no current therapies that have been shown to alter the course of the disease and the rate of decline in renal function [3].

ADPKD is caused by mutation of one of two genes, *PKD1* or *PKD2* (MIM 601313 and 613095). Other rare genetic causes for multiple renal cysts are well recognised but are typically clinically distinct from ADPKD [4]. Initial studies suggested that approximately 85% of cases of ADPKD are due to *PKD1* mutations with the remainder in *PKD2*[5]. However several recent studies have suggested that *PKD2* mutations may be present in up 36% of cases depending on the population screened (Table 1). These studies have also suggested that in many individuals, *PKD2*-linked disease is mild and they either do not present or come late to medical attention [2,6]. This is supported by studies reporting that patients with *PKD2* mutations develop ESRF some 15–20 years later than those with a *PKD1* mutation (69 yrs vs 53 yrs respectively) [7,8]. As further clinically useful within-gene genotype-phenotype correlations do not exist, this genic effect is one of few major predictors of clinical severity in ADPKD [9]. Clinical indicators of disease severity such as magnetic resonance-measured renal volume have been proposed as important, both in monitoring early progression and in providing more accurate long term predictions of outcomes such as ESRF before a decline in GFR is evident, but are currently confined to research use [10,11].

**Table 1:** **Detection rate of *PKD2 *mutations in different published studies**

**Study**	**% cases with*PKD2*mutation**	**Cohort**
J Am Soc Nephrol 18:SA-PO93, 2007	36	Olmsted County population study
Barua et al. 2009 [25]	26	Single centre, ESRF excluded
Peters et al. 1992 [5]	15	Multi-centre, ADPKD kindreds
Rossetti et al. 2007 [12]	15	Multi-centre, CRISP study (GFR >70 ml/min)
Rossetti et al. 2003 [26]	12	Multi-centre, ADPKD with vascular phenotype
Garcia-Gonzalez et al. 2007 [22]	15	Multi-centre, ESRF included
This study	20	Single centre, CKD5 and ESRF excluded

*PKD* gene testing has recently been approved for clinical use in the UK by the UK Genetic Testing Network (http://www.ukgtn.nhs.uk) and is available in other countries worldwide. To provide improved prognostic and genetic counselling information, we introduced *PKD2* mutation testing into a hospital nephrology outpatient service that sees pre-ESRD patients, as a clinical tool to assign patients and their families to either *PKD2* or non-*PKD2* groups. *PKD2* testing has the potential to provide accurate genotype information for an individual without the need to collect additional family members for linkage analysis or to undertake the more complex and technically challenging *PKD1* mutation testing [12,13]. ADPKD patients attending a single nephrology outpatient clinic formed the study cohort, which excluded those already referred to a low clearance clinic (CKD5) or already receiving RRT. Here we show that direct *PKD2* mutation testing offers a means of providing additional prognostic information to individuals with ADPKD and their families especially when used with a detailed family history.

## Methods

### Clinical assessment

Sequential unrelated adults referred to the Cambridge Renal Genetics and Tubular Disorders Clinic (http://www.cuh.org.uk/addenbrookes/services/clinical/renal/services/renal_genetics_tubular_disorders_clinical.html) between 2005 and 2009 with, or at risk of, a primary clinical diagnosis of ADPKD were included in this study. Patients attending other clinics such as low clearance (CKD5, eGFR ≤ 15 mL/min/1.73 m^2^) or renal replacement/transplant clinics were excluded. Standard diagnostic ultrasound criteria were used if there was a known family history of ADPKD [14]. If no family history was available, the diagnosis of ADPKD was made if renal imaging demonstrated bilateral nephromegaly with multiple cortical and medullary cysts with or without hepatic/pancreatic cysts, and where other diagnoses associated with bilateral renal cysts were excluded. Clinical and family data was obtained from index cases during their routine clinical assessment. Additional family data, where necessary, was obtained from medical records with full written consent. The following demographic and clinical data were collected: age, gender, blood pressure, antihypertensive treatment, serum creatinine/eGFR at presentation, indications for renal ultrasound, current renal function, initiation of dialysis and/or death, number of affected relatives and age of their ESRF. Hypertension was defined as a blood pressure > 140/90 mmHg on more than one occasion, or regular prescription of antihypertensive medication. Renal volume was not routinely evaluated.

This study was approved by the Cambridge Central Research Ethics Committee (project number 08/H0306/62) and registered for audit activity with Cambridge University Hospitals NHS Foundation Trust.

### Mutation detection

Genomic DNA from all index cases was extracted by standard methods and screened for *PKD2* mutations by exon-specific PCR and direct sequencing. Further details of the methods used are available in Additional file 1: Table S1 or via the UKGTN website (http://www.ukgtn.nhs.uk/gtn/Home). *PKD2* sequence variants were defined as pathogenic if either previously reported or destructive to the integrity of the encoded protein, and if mis-sense, as likely pathogenic/likely neutral using the criteria described in the PKD Mutation database (http://www.pkdb.mayo.edu).

### Statistical analysis

All data are presented as mean ± SE or median (IQR) as appropriate. Comparisons of proportions were made with Fisher’s Exact or Mann Whitney *U* test. Continuous variables were compared with the Student’s *t*-test or Wilcoxon Rank Sum test as appropriate. A p-value of <0.05 was considered significant. Patients were included in analyses of renal function if their creatinine values were available for two or more time points. Rate of decline in renal function was assessed using response feature analysis [15], with the regression slope of eGFR over age as the response feature. CKD stages 3–5 were defined as the first time point when eGFR reached standard threshold criteria on three consecutive measurements. Time to onset of CKD was assessed using a Cox proportional hazards model, with PKD genotype as the predictor variable. The proportional hazard assumption was assessed using scaled Schoenfeld residuals [16]. For building proportional hazard models, goodness of fit was assessed by estimating the empirical Nelson-Aalen cumulative hazard function with the Cox-Snell residuals as the time variable, along with the censoring variable [17].

All primary analyses using regression models were performed using diagnostic sub-type as the only predictor variable. All analyses were performed using Stata SE v11.2, (College Station, Texas).

## Results

### *PKD2* mutation detection

**Figure 1:**
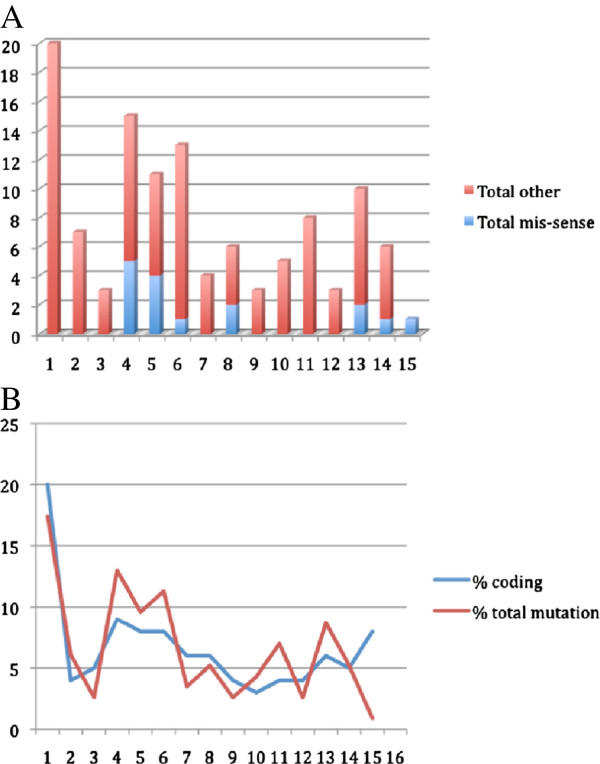
**Indications for ultrasound screening at time of diagnosis as given by clinician. Number of subjects in each category are shown.** Total non-*PKD2* = 115; *PKD2* = 27. UTI = urinary tract infection; SAH = subarachnoid haemorrhage.

142 individuals with ADPKD were studied. We identified *PKD2* mutations in 27 (19%). Identified sequence variants are listed in Table 2. Twenty-five different pathogenic or likely pathogenic *PKD2* mutations were identified in 27 cases, with an additional one being indeterminate according to the PKD Mutation Database. Eight of these were nonsense (31%), 11 frameshifting (42%; 6 small insertions/deletions and 5 splice site alterations), 5 were substitutions (19%), and 1 whole gene deletion was found (4%). Nine (35%) had been previously described in the PKD Mutation database. The mutations identified more than once were all found in unrelated probands. The whole gene deletion was initially identified by FISH following the previous identification of a presumed balanced translocation within the family (46,XX,t(4;11)(q22;q21); manuscript in preparation). Its significance was confirmed by array CGH and MLPA showing haploinsufficiency for *PKD2* that segregated in all family members with ADPKD (data not shown). Combining the 27 mutations identified in this study with the 115 pathogenic mutations listed in the PKD database with this study (20% of the total), Figure 1 shows the proportion of mis-sense and other mutations in each exon, and their distribution along the encoded protein. No mutation hotspots were apparent. The majority (87%) are predicted to be inactivating, in keeping with previous studies [18]. In addition, 4 neutral amino acid substitutions (two novel) were found in eight individuals (Table 2). These subjects were classified as 'non-*PKD2*'.

**Table 2:** **Sequence variants identified in *PKD2***

**Exon**	**Mutation designation**	**cDNA change**	**Amino acid change**	**Mutation type**	**Significance**	**No. of cases**
1	E95X	c.283 G > T	Glu95X	nonsense	pathogenic	
1	P150L	c.449 C > T	Pro150Leu	missense	likely neutral	
1	A190T	c.568 G > A*	Ala190Thr	missense	likely neutral	4
1	305_307dupAGG	c.305_307dupAGG	Val 103 fs	frameshift	pathogenic	
1	397del44	c.397del44	Ser133fs	frameshift	pathogenic	
1	401_410delTGGGCGCGCG	c.401_410delTGGGCGCGCG	Val134fs	frameshift	pathogenic	
2	W201X	c.602 G > A*	Trp201X	nonsense	pathogenic	2
2	R213X	c.637 C > T	Arg213X	nonsense	pathogenic	2
IVS2	IVS2 + 5insA	c.709 + 5insA	Leu237fs	splice	likely pathogenic	
IVS2	IVS2-2A > G	c.710-2A > G*	Leu237fs	splice	pathogenic	
4	R322Q	c.965 G > A*	Arg322Gln	missense	pathogenic	
4	R361X	c.1081 C > T*	Arg361X	nonsense	pathogenic	
IVS4	IVS4 + 1 G > A	c.1094 + 1 G > A*	Ala365fs	splice	pathogenic	
5	G390V	c.1169 G > T	Gly390Val	missense	pathogenic	
6	F482C	c.1445 T > G*	Phe482Cys	missense	likely neutral	2
6	W507X	c.1521 G > A	Trp507X	nonsense	pathogenic	
7	1668dupA	c.1668dupA	Gln557fs	frameshift	pathogenic	
IVS8	IVS8 + 5 G > C	c.1898 + 5 G > C	Leu573fs	splice	pathogenic	
IVS8	IVS8 + 1 G > A	c.1898 + 1 G > A*	Leu573fs	splice	pathogenic	
8	Q613X	c.1837 C > T	Gln613X	nonsense	pathogenic	
8	C632Y	c.1895 G > A	Cys632Tyr	missense	likely pathogenic	
10	2085_2087delAGCinsGG	c.2085_2087delAGCinsGG	Lys695fs	frameshift	pathogenic	
11	2163dupC	c.2163dupC	Val722fs	frameshift	pathogenic	
11	R730Q	c.2189 G > A	Arg730Gln	missense	likely neutral	
11	R742X	c.2224 C > T*	Arg742X	nonsense	pathogenic	
13	R807Q (a)	c.2420 G > A*	Arg807Gln	missense	indeterminate	
14	R845X	c.2533 C > T*	Arg845X	nonsense	pathogenic	
14	L867P (a)	c.2600 T > C	Leu867Pro	missense	likely pathogenic	
15	D919N	c.2755 G > A	Asp919Asn	missense	likely pathogenic	
1-15	EX1_EX15del			deletion	pathogenic	

* = mutations already described in the PKD mutation database (http://www.pkdb.mayo.edu) (a) = both variants found in the same patient.

One patient harbouring a mis-sense mutation had a second, indeterminate change, R807Q (Table 2). Although his mother was clinically affected, parental samples were not available for segregation analysis.

### Clinical Characteristics

**Figure 2:**
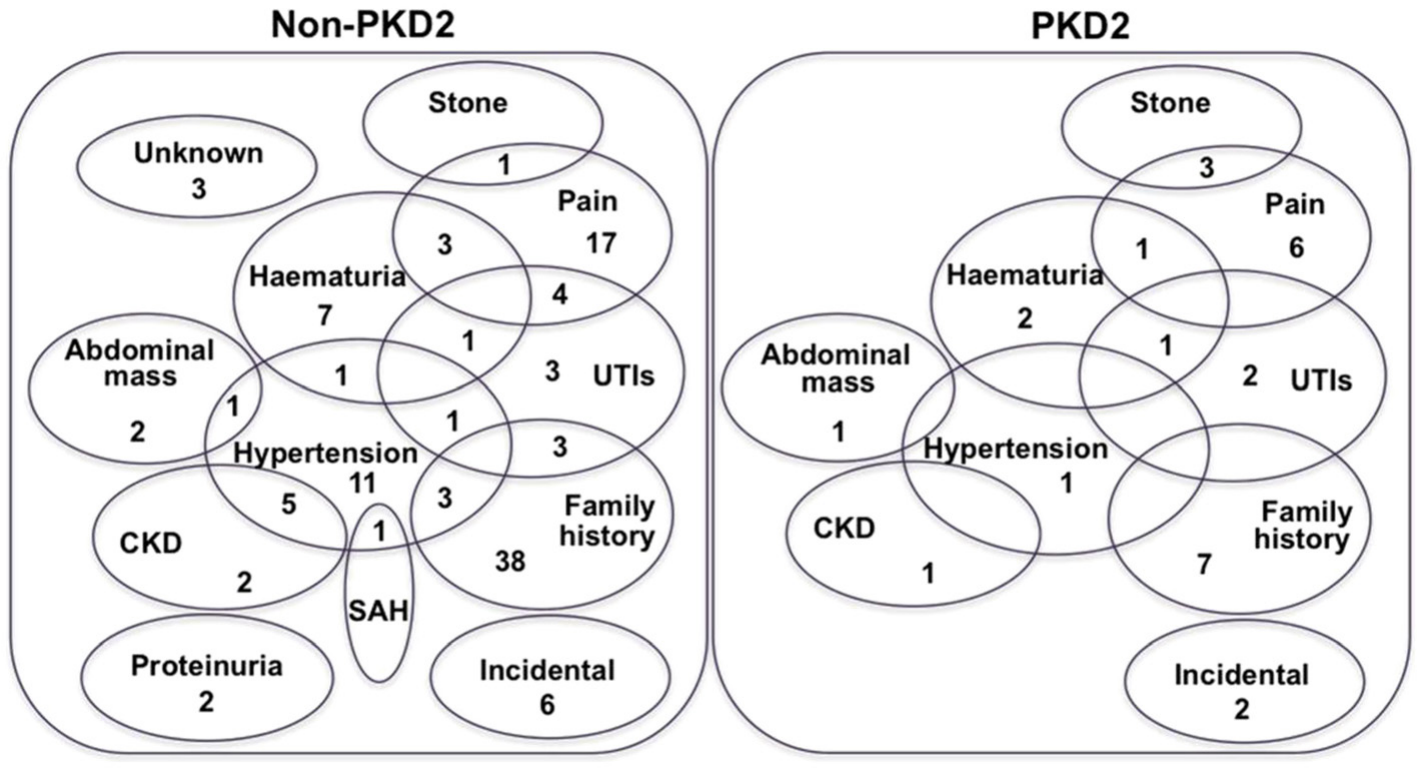
**(A) Distribution of mutations along the *PKD2 *coding sequence.** Mutations described in this report have been combined with pathogenic mutations identified from the PKD mutation database. Other = non-sense, frame-shift and insertion/deletion mutations). (**B**) Percentage of total *PKD2* mutations (red); each exon's percentage of the total coding sequence (blue).

Those with ('*PKD2'*) and those without ('non-*PKD2'*) a *PKD2* mutation were grouped for further analysis. Indications for initial diagnostic abdominal ultrasonography are given in Figure 2, and clinical characteristics in Table 3. There was no significant difference between the *PKD2* and non-*PKD2* groups when comparing range of clinical presentations, gender ratio, mean age at diagnosis, proportion with hypertension before or during follow-up, or serum creatinine/eGFR at clinic presentation (Table 3). Renal volume data were not available.

**Table 3:** **Characteristics of ADPKD patients screened for *PKD2 *mutations. ns = not significant**

	**Non-*PKD2***	***PKD2***	***p-*value**
Number (%)	115 (80.3)	27 (19.7)	
Male:female	0.59	0.59	
Age at clinic presentation y	49.0 ± 13.3	49.5 ± 14.8	ns
Age at diagnosis y	37.4 ± 15.4	42.6 ± 14.4	ns
Hypertensive at diagnosis %	42.6	40.7	ns
Treatment for hypertension %	84.3	70.4	ns
Progression to ESRF %	12.2	0	*p* < 0.03
eGFR at presentation ml/min/1.73 m^2^	67.0 ± 27.6	74.0 ± 28.4	ns
No FH of ADPKD %	35.6	25.9	ns
FH with ESRF %	50.4	40.7	ns
FH with no ESRF %	13.9	33.4	*p* < 0.03
Median age of family member at ESRF y (range)	54.1 (33–83)	65.5 (50–86)	*p* < 0.0001
Number with FH ESRF < 50 yrs	31	0	
Number with FH ESRF < 60 yrs	69	4	
Number with FH ESRF < 70 yrs	81	9	
Number with FH ESRF ≥ 70 yrs	8	6	
Number of family members with ESRF (where age known)	89	15	

**Figure 3:**
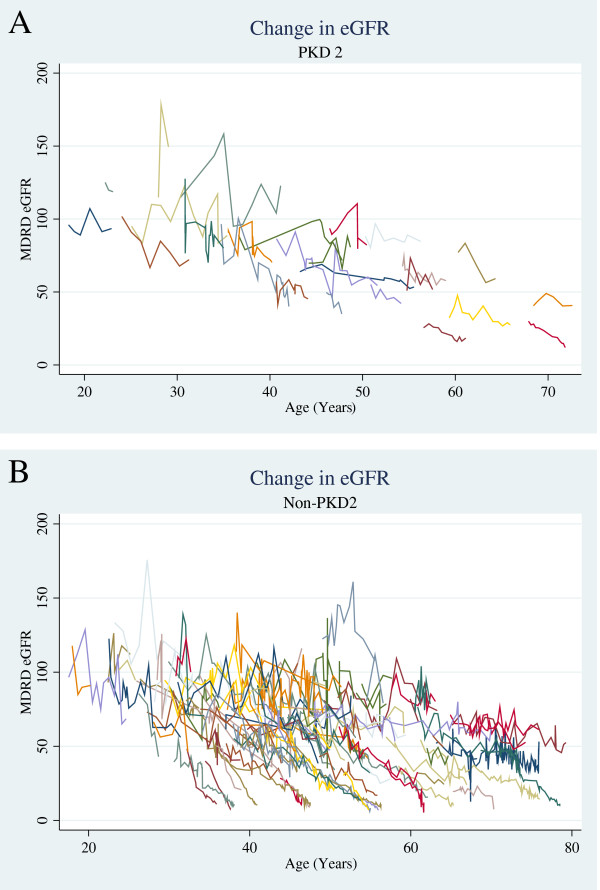
**Change in eGFR values over time (age in years) for individuals with (A) and without (B) a *PKD2 *mutation.**

**Figure 4:**
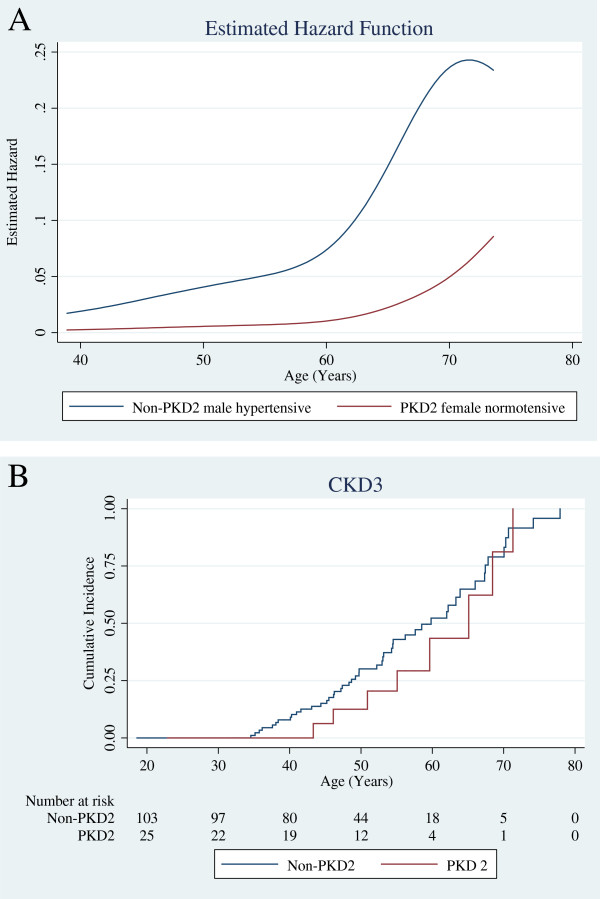
**Likelihood of development of CKD3 according to age and genotype** (**A**) The hazard function estimates the event rate at a given age, conditional on event-free survival to that age. The greatest and smallest estimated hazard of developing CKD3 was associated with non-PKD2 male hypertensive subjects and PKD2 female normotensive subjects. Although this is congruent with point estimates from a multivariate Cox proportional hazards model (Additional file 2: Table S2), these factors did not reach statistical significance in this cohort. (**B**) Kaplan-Meier estimates of time to CKD3.

**Figure 5:**
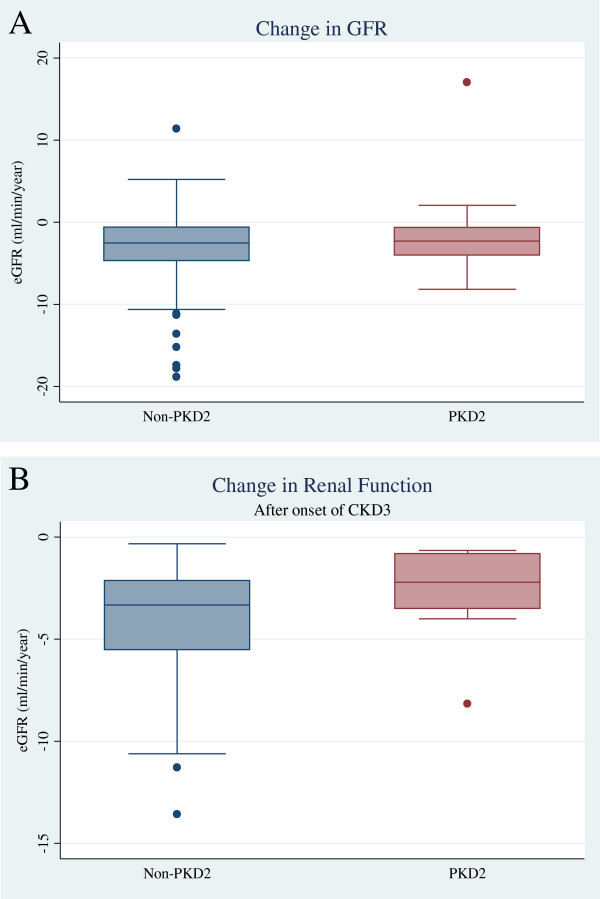
Box plots (median, lower and upper quartiles and range) of rate of change in eGFR during follow-up: (A) according to genotype and (B) after onset of CKD3.

Serial renal function data were available for 130 of the original 142 cases, a similar percentage in each group. A total of 2103 creatinine values were recorded for 25 *PKD2* and 105 non-*PKD2* mutation carriers, with a median 12 (6–22) values per patient, representing renal outcome data for 48.3 patient years for *PKD2* and 49.5 patient years for non-*PKD2* (Figure 3). No *PKD2* patient progressed to ESRF during follow-up, compared to 14 (13%) of the non-*PKD2* group. In addition, 20/105 (19%) non*-PKD2* vs. 1/25 (4%) *PKD2* patients had, or developed, at least CKD3 under the age of 40 years (*p* = 0.08). For the whole group the proportion developing CKD3/4 was not significantly greater within the non-*PKD2* group (Table 4), and the median age at development of CKD3 was not dependent on *PKD2* mutation status. Absence of a *PKD2* mutation was associated with a non-significant trend towards a greater hazard of developing CKD3, (HR 1.45; 0.68-3.07, *P* = 0.33, Figure 4) even when adjusting the model for gender and hypertension (HR 1.54; 0.73-3.29, *P* = 0.26) (Additional file 2: Table S2). Once CKD3 had developed, the rate of decline in renal function (ml/min/year) for non-*PKD2* and *PKD2* cases was similar (non-*PKD2:* 3.3 (2–5.5), *PKD2:* 2.2 (0.8 - 3.5), *p* = 0.1 (Figure 5)).

**Table 4:** By-category comparison between genotypes of proportion of cases developing CKD

		**Proportion**	**%**	***p*-value**
At least CKD3	Non-*PKD2*	48/105	46	0.15
	*PKD2*	8/25	32	
At least CKD4	Non-*PKD2*	23/105	22	0.21
	*PKD2*	3/25	12	
CKD5 or RRT	Non-*PKD2*	14/105	13	0.04
	*PKD2*	0/25	0	

The number of patients reporting no known family history of ADPKD did not differ significantly between genotypes (36% of non-*PKD2* and 26% of *PKD2*, Table 3); neither did the occurrence within the family of ESRF (Table 3). However, patients with a *PKD2* mutation were more likely to have a FH featuring preservation of renal function (33.4% vs 13.9%, p < 0.03) and ESRF occurring at an older age in affected relatives (65.5 ± 10.2 yrs vs 54.1 ± 9.8 yrs, p < 0.0001). No patient with a FH of ESRF at age < 50 years had a *PKD2* mutation (PPV 100%, sensitivity 0.71). In contrast, a FH of ESRF that did not occur until at least 70 years was predictive of a *PKD2* mutation (PPV 88%, sensitivity 0.29).

## Discussion

This preliminary, single centre, small scale prospective study was designed to evaluate the use of routine *PKD2* mutation testing in a nephrology outpatient clinic. The clinic does not see those who have already developed ESRF. Since it is known that *PKD2* status identifies those with a milder clinical phenotype and delayed progression to ESRF by up to 20 years [7], this information would be valuable to provide patients and their families with improved prognostic information in the earlier stages of their disease and to determine whether there were other simple, patient-reported variables that could be used to predict genotype and prognosis. Therefore additional information about *PKD1* mutation status was not required, as assignment to the *PKD2* group did not require this. Similarly, the role of reduced-penetrance *PKD1* alleles on the expression of *PKD2*-linked disease is not known and has not been evaluated in other studies [19,20]. Patients carrying both *PKD1* and *PKD2* pathogenic mutations are also very rare [21].

We demonstrated that a fifth of an unselected ADPKD patient cohort from a single clinic that sees patients only with eGFR > 15 mL/min/1.73 m^2^ had a likely pathogenic *PKD2* mutation. Our patient characteristics are very similar to those reported by Garcia-Gonzalez et al. (2007), although the number reaching end-stage was lower in our study (10% vs. 21%) [22]. In its design, our study was likely to favour a higher *PKD2* detection rate, as testing was not offered to individuals with CKD5 or receiving RRT. In addition, defining the proportion of all patients with ADPKD harbouring a *PKD2* mutation was not a primary aim of this study. Consistent with this, other studies have shown a detection rate of ~15% when all prevalent cases have been included. Therefore the conclusions of this study can only be applied to similar clinical cohorts. As patients with ADPKD and CKD1-4 are typically seen in the nephrology out-patient clinic setting that does not include those with CKD5 and ESRF, this should not present significant clinical difficulty.

Interestingly, there appeared to be no overall difference between the two groups in the risk of developing, or age of development of, CKD3 or CKD4, or in the rate of functional deterioration (eGFR) once CKD had supervened. It is unclear whether the former was due to the cohort size or selection of patients with a milder phenotype due to the exclusion of those that presented with CKD5. Post-hoc power calculations demonstrated that the sample had 60% power to detect a renal survival difference for CKD3. For 80% power a sample size of 169 patients would be required (34 with a *PKD2* mutation and 135 without). Therefore the relatively small sample size will limit some of the inferences that can be drawn from our data. Although the proportion of patients developing CKD3/4 in the two groups were not statistically different, we recognise that our study was sub-optimally powered to detect such a difference, but the direction of the effect of non-PKD2 status would be consistent with previous reports of worse renal outcomes in this patient group. There was greater heterogeneity in the renal phenotype of non-*PKD2* patients, with a minority experiencing early-onset CKD (Figure 3). Further well- powered longitudinal studies in the same clinical setting, using multi-centre registries of genotyped patients, are therefore required to address this including the contribution of other factors such as age, gender, hypertension and albuminuria.

Currently, genic variation in ADPKD is the main factor predicting renal outcome, ESRF occurring up to 15–20 years later in patients with a *PKD2* mutation when compared to those with a *PKD1* mutation [7]. MRI imaging data from the CRISP study suggests that this is due to a lower number of cysts being present in *PKD2*-related disease, rather than differences in the rate of cyst growth or renal enlargement [23,24]. Although not generally in clinical use, measurement of single or serial renal volumes may better predict disease severity, with larger kidneys predicting a more rapid rate of decline in GFR [23]. However, routine *PKD2* mutation testing that does not require the patient to be present, will become more economical and identifies a group of patients likely to have preserved renal function for longer [18].

The use of direct mutation testing rather than linkage analysis also has the benefit of being available to individuals rather than extended families. About a third of our cohort was not aware of a FH of ADPKD at the time of clinic attendance and would therefore not have been suitable for linkage analysis. This most likely represents a combination of factors including new mutation, undiagnosed disease in relatives and unrevealed diagnoses rather than a higher than expected new mutation rate alone. Whilst the lack of a FH means that the Ravine criteria for the diagnosis of ADPKD cannot be strictly employed it is likely that other causes of PKD, such as renal cysts and diabetes (RCAD, OMIM 137920) which can phenocopy ADPKD, were not sufficiently common to account for the discrepancy with the published data. Similarly the role of hypomorphic alleles in modifying disease presentation remains unknown although is unlikely to account for significant non-penetrance. In the study by Barua et al. [25], the new mutation rate determined following parental ultrasound examination was ~10%, suggesting that in more than a fifth of our patients, a diagnosis of ADPKD is not being communicated or is undiagnosed. Wider use of parental screening after appropriate counselling is therefore justified to clarify disease risks for family members. Further work will be required to explore how this may be more generally implemented in non-specialist clinics and in primary health care.

The detection rate of ~ 20% *PKD2* mutations is in broad agreement with other studies (range 15-36%; Table 1) [5,6,12,13,22,25,26]. However, each of these studies employed different inclusion and exclusion criteria and sampled different populations, making ascertainment bias likely. The estimate of 36% in one study may represent the true frequency when population screening for ADPKD is carried out, and would likely represent the ascertainment of asymptomatic, undiagnosed and therefore mild cases.

Unlike direct *PKD1* mutation testing, where it remains difficult to assign pathogenicity to some variants, the majority of *PKD2* sequence variants can be assigned as likely or highly likely to be pathogenic [12,13]. Sensitivity of mutation detection is therefore likely to be very high when using direct sequencing, although this needs to be formally tested in families where linkage data are also available [25]. In the study by Veldhuisen et al. (1997), mutations were found in 57% of 35 families linked to the *PKD2* locus when 80% of the gene was screened by single strand conformation polymorphism analysis and direct sequencing [27]. With newer methods and complete gene sequencing and dosage analysis, a higher detection rate is likely. Our figure of ~ 20% may therefore still slightly underestimate the true number of *PKD2* mutations in this cohort of patients with CKD1-4. Dosage analysis is now available and should be included in future studies. However, the PKD Mutation Database suggests that large deletions and duplications in *PKD2* are rare. Only a single deletion among 115 pathogenic *PKD2* mutations reported here and in the database would have been missed without dosage analysis. Thus a > 98% detection rate by screening coding exons and their splice sites can be achieved. This represents the best current method for identifying individuals and families that harbor a *PKD2* mutation and may therefore have a better prognosis.

Although molecular testing for ADPKD is now available in the UK (http://www.ukgtn.org.uk), it has not been routinely used in nephrology out-patient clinics for diagnostic, predictive or prognostic testing. At present, diagnosis and family screening are almost exclusively carried out using imaging combined with a FH. The main utility of genetic testing to date has been in research to define the natural history of the disease by genotype, and to identify genotype/phenotype correlations [26]. Indications for clinical molecular testing include establishment of the correct diagnosis if there is no family history and if there are atypical features on imaging; exclusion testing for potential living-related donors and prenatal/pre-implantation genetic diagnosis [28]. Of these, the ~ 20% detection rate of *PKD2* mutations is likely to be most clinically useful in screening potential living-related donors, particularly for those with normal ultrasounds below age 40 [14]. Non-*PKD2* status is assumed here to represent *PKD1*-linked disease, i.e. in this context the test is 100% specific and sensitive.

Of the clinical variables recorded in this study, few had significant predictive power to identify patients likely to have a *PKD2* mutation. In the multivariate model adjusting for gender and hypertension, point estimates suggested that non-*PKD2* disease, male gender and the presence of hypertension may together be associated with earlier onset of CKD3 (Additional file 2: Table S2), although formal significance levels were not reached in this cohort due to its size. However, the directional nature of the effect supports previous reports indicating a milder renal phenotype with *PKD2* mutations [18].

The lack of difference in progression from CKD3 between the two groups was unexpected, but may be due to a number of factors. Firstly, although 16 non-*PKD2* cases had developed CKD4 earlier than the youngest *PKD2* case with CKD4 (59.6 years), 25% (5/20) of non-*PKD2* cases had not yet reached CKD3 by the age of 62, compared to 17% (1/6) of *PKD2* cases, further highlighting that likely *PKD1* mutations may be associated with mild disease [20]. Secondly, our analysis may underestimate progression of CKD in non-*PKD2* cases due to ascertainment bias through exclusion of patients with existing CKD5/ESRD, since our results suggest a predominance of non-*PKD2* in patients in this group. Thirdly and most likely, our available sample may be underpowered to detect differences in progression to CKD3. This further emphasises the continued need to conduct long-term studies on large, genotyped cohorts.

However, the comparable rate of decline in eGFR after the onset of CKD3 between the two groups is similar to that observed in previous studies where no significant differences between genotypes were found, suggesting that the underlying disease process is independent of the causal mutation [23]. This is also supported by previously observed similar rates of change in renal volume between *PKD1* and *PKD2* groups, although the *PKD2* group have fewer cysts and smaller kidneys at a given age [24].

The predictive value of the FH was strong [25]. *PKD2* patients were more likely to have a FH of ADPKD without progression of CKD, and the average age of a family member with ESRF was significantly higher than the non-*PKD2* group (Table 3). This reflects the ascertainment of *all* degrees of disease among family members and is in keeping with the previously reported milder clinical phenotype of *PKD2*-linked disease [7]. A FH of ESRF before age 50 was highly predictive of non-*PKD2* status (PPV 100%, sensitivity 0.71). Therefore, in PKD patients with relatively preserved renal function, the disease course of affected family members can usefully be used to direct genetic testing, with *PKD2* testing targeted to those patients with a FH of ESRF occurring only over 50 yrs. Similarly, a FH of ESRF occurring at or after age 70 was strongly predictive of a *PKD2* mutation (PPV 88%, sensitivity 0.29). However, sensitivity was low, as some individuals with apparent *PKD1*-linked disease (non-*PKD2*) can have very mild disease [29], as observed in our cohort (Figure 3). Our results confirm those of Barua *et al.* who found that the presence of at least one family member who developed ESRF before 55 years was highly predictive of *PKD1* (positive predictive value 100%, sensitivity 72%) [25]. Having at least one family member with ESRF after 70 years of age was also highly predictive of *PKD2* in that study. FH alone has limitations if additional family information is not available, or if an individual represents a new mutation (i.e. if both parents have normal renal imaging). Such patients are suitable for genetic analysis.

Importantly, a positive *PKD2* test allows other family members to consider predictive genetic testing. Therefore, whilst a detailed FH has some predictive value and patients with a FH of ESRF before age 50 can be excluded from *PKD2* testing, genotyping offers the best means to provide prognostic information in the absence of other discriminant clinical parameters. In summary, we suggest that *PKD2* screening can be routinely offered to ADPKD patients except where they or their family members have developed ESRF under age 50. Additional studies in larger cohorts or using pooled data analysis would also be of value in refining the current indications and criteria for PKD gene testing.

## Conclusions

In this study we have used mutation analysis to identify a group of patients with pathogenic *PKD2* mutations but preserved renal function. *PKD2* mutations have previously been reported to confer substantially better renal survival compared to *PKD1*-linked disease, making this an important prognostic marker. We have shown that in a population of patients with CKD1-4, *PKD2* testing was positive in about a fifth of patients. When used alone, it could not discriminate those individuals with milder renal disease defined by stage of CKD, but did identify a group less likely to progress to ESRF. Combined with FH, it offers useful prognostic information for individuals and their families, and can be routinely offered to all but those whose relatives have developed ESRF before the age of 50.

## Abbreviations

CGH: Comparative genomic hybridisation; ADPKD: Autosomal dominant polycystic kidney disease; ESRF: End-stage renal failure; CKD: Chronic kidney disease; GFR: Glomerular filtration rate; eGFR: Estimated glomerular filtration rate; RRT: Renal replacement therapy; IQR: Interquartile range; MLPA: Multiplex Ligation-dependent Probe Amplification; FISH: Fluorescence *in situ* hybridization; MRI: Magnetic resonance imaging.

## Competing interests

The authors declare that they have no competing interests.

## Authors’ contributions

RNS and FEKF conceived the study and participated in its design and coordination. CR, DS, FEKF and RNS reviewed patients and collected clinical data. SW and LD carried out the molecular genetic studies. CR and TFH performed the statistical analysis. RNS, CR and FEKF drafted the manuscript. All authors read and approved the final manuscript.

## Authors’ information

FEK is Professor of Nephrology at the University of Cambridge and honorary consultant in renal medicine. RNS is a Reader in Renal Genetics at the University of Cambridge and honorary consultant in medical genetics.

## Pre-publication history

The pre-publication history for this paper can be accessed here:

http://www.biomedcentral.com/1471-2369/13/79/prepub

## Supplementary Material

Additional file 1 Table S1.PCR primers used in *PKD2* mutation analysis. PCR conditions were 96°C for 3 mins followed by 33 cycles of 96°C for 30 secs, 60°C for 1 min, 72°C for 1 min and a final step of 72°C for 5 mins. Additional information available on request (http://rns13@cam.ac.uk). Click here for file

Additional file 2 Table S2.Hazard ratio of developing CKD3 adjusted for gender and hypertension. where pkd1 = non-*PKD2*; 1.sex = male gender; 1.htnbin = a diagnosis of hypertension. Click here for file
