# Supplementary material for: Clinical utility of PKD2 mutation testing in a polycystic kidney disease cohort attending a specialist nephrology out-patient clinic
Source: BMC Nephrol. 2012 Aug 3;13:79. doi: 10.1186/1471-2369-13-79 (PMC3502417; doi:10.1186/1471-2369-13-79)
Supplement: Additional file 1 — Table S1.PCR primers used in PKD2 mutation analysis. PCR conditions were 96°C for 3 mins followed by 33 cycles of 96°C for 30 secs, 60°C for 1 min, 72°C for 1 min and a final step of 72°C for 5 mins. Additional information available on request (http://rns13@cam.ac.uk). [file 1471-2369-13-79-S1.docx]

| **EXON** | **FORWARD PRIMER** | **LOCATION** | **FORWARD PRIMER SEQUENCE** | **REVERSE PRIMER** | **LOCATION** | **REVERSE PRIMER SEQUENCE** | **PRODUCT SIZE** |
| --- | --- | --- | --- | --- | --- | --- | --- |
|  |  |  | *** M13F** TGT AAA ACG ACG GCC AGT |  |  |  |  |
| **1A1** | **PKD2_1_A1_F*M13F** | **PKD/1.A1** | GCC GGG AAG AAA GGA ACA TGG | **PKD2_1_A1_R** | **PKD/1.A2** | TGC GCT GCA TCT CGA TCT CC | 260 |
| **1A2** | **PKD2_1_A2_F*M13F** | **PKD/1.F3** | CGT GCA GCC TCA GCA G | **PKD2_1_A2_R** | **PKD/1.F4** | GAG AGC GGA GGA GAA GG | 222 |
| **1B** | **PKD2_1_B_F*M13F** | **PKD/1.A3** | GCC TGG AGA TCG AGA TGC | **PKD2_1_B_R** | **PKD/1.A4** | GCC GTG GTA GCC CCC AAG | 305 |
| **1C1** | **PKD2_1_C1_F*M13F** | **PKD/1.A5** | GAG GTG GAA GGG GAA GAA GGC G | **PKD2_1_C1_R** | **PKD/1.A6** | CTT CCA GGG GGA GGT GGC G | 241 |
| **1C2** | **PKD2_1_C2_F*M13F** | **PKD/1.F5** | CCG GCG AGA GGA CCA GGG | **PKD2_1_C2_R** | **PKD/1.F6** | GTT CTG GTT CGT GCA TCT GCC GCT | 174 |
| **2** | **PKD2_2_1_F*M13F** | **PKD/1.C1** | GAA TCT CCC TTA TAG GTG AAC | **PKD2_2_1_R** | **PKD/1.C2** | TGG ATA GGT CAA ATC TTT TCA | 280 |
| **3** | **PKD2_3_1_F*M13F** | **PKD/1.C3** | CTG CTG GTA TGT GAA TGT GT | **PKD2_3_1_R** | **PKD/1.D8** | AAC AAT TTT CTG TGA TAG AGA GG | 250 |
| **4** | **PKD2_4_1_F*M13F** | **PKD/1.C5** | ATA GAG TTG CCA AAT GCT TG | **PKD2_4_1_R** | **PKD/1.C6** | GAA TGA ATA TCA CCG AGT GG | 390 |
| **5** | **PKD2_5_1_F*M13F** | **PKD/1.C7** | CCT CAA GTG TTC CAC TGA TT | **PKD2_5_1_R** | **PKD/1.C8** | GTA GCT AAC TGC AGG CAA AG | 370 |
| **6** | **PKD2_6_1_F*M13F** | **PKD/1.C9** | CTG GCT GTA TTC ATG TGT TG | **PKD2_6_1_R** | **PKD/1.D1** | AAT GCT GAG GAG ATC AAA GA | 410 |
| **7** | **PKD2_7_1_F*M13F** | **PKD/1.C4** | TCG GGT AAG TAT AAT GGT GAG C | **PKD2_7_1_R** | **PKD/1.E1** | TTC AAG TAT TCC ATG ATT TTG TGG | 413 |
| **8** | **PKD2_8_1_F*M13R** | **PKD/1.E2** | CAT CCA TGT TGT AAC CTG TCA GA + M13R | **PKD2_8_1_R*M13F** | **PKD/1.E3** | TGG TGG TCA TAT AGC AAC CTC A +M13F | 431 |
| **9** | **PKD2_9_1_F*M13F** | **PKD/1.D6** | GCA TCA ACT AGT GGA CAT TC | **PKD2_9_1_R** | **PKD/1.D7** | GAG AAG ACA AGG ATT TAC GAA G | 240 |
| **10** | **PKD2_10_1_F*M13R** | **PKD/1.E4** | GGA TAA ACA AAA AGG CAT GTG TC + M13R | **PKD2_10_1_R*M13F** | **PKD/1.E5** | TCT GGG TGA AAC AAT GCT CA + M13F | 295 |
| **11** | **PKD2_11_1_F*M13F** | **PKD/1.A9** | AAA CAG ATG CAA AAG GAG AA | **PKD2_11_1_R** | **PKD/1.B1** | AGA AGC AGG AAT TTT TCA GA | 230 |
| **12** | **PKD2_12_1_F*M13F** | **PKD/1.E6** | CTG TGT TGA GGG TGA ACT GG | **PKD2_12_1_R** | **PKD/1.E7** | TGT TTG ATA CAT CTG TGG TGT TG | 281 |
| **13** | **PKD2_13_1_F*M13F** | **PKD/1.E8** | GCC CAA GTC CTT GGT GAG | **PKD2_13_1_R*M13R** | **PKD/1.E9** | GGA ACT GCC TGG TCT CAT GT + M13R | 335 |
| **14** | **PKD2_14_1_F*M13F** | **PKD/1.F1** | AGC GGC ATC CGA GAG TTA AT | **PKD2_14_1_R*M13R** | **PKD/1.F2** | TTC AAA TAC AAC TGT CAG CAA CA + M13R | 431 |
| **15** | **PKD2_15_1_F*M13F** | **PKD/1.B8** | CCA GCC TTA CCA AAC TAC AG | **PKD2_15_1_R** | **PKD/1.B9** | GTG CTT GTT ACA GCA ATT CA | 400 |

Table S1.

PCR conditions were 96^o^C for 3 mins followed by 33 cycles of 96 ^o^C for 30 secs, 60 ^o^C for 1 min, 72 ^o^C for 1 min and a final step of 72 ^o^C for 5 mins. Additional information available on request.
