# Supplementary material for: Clinical utility of PKD2 mutation testing in a polycystic kidney disease cohort attending a specialist nephrology out-patient clinic
Source: BMC Nephrol. 2012 Aug 3;13:79. doi: 10.1186/1471-2369-13-79 (PMC3502417; doi:10.1186/1471-2369-13-79)
Supplement: Additional file 2 — Table S2.Hazard ratio of developing CKD3 adjusted for gender and hypertension. where pkd1 = non-PKD2; 1.sex = male gender; 1.htnbin = a diagnosis of hypertension. [file 1471-2369-13-79-S2.docx]

| _t | HR | SE | z | P>\|z\| | 95% CI | |
| --- | --- | --- | --- | --- | --- | --- |
| Non-PKD2 | 1.54 | 0.59 | 1.13 | 0.26 | 0.73 | 3.29 |
| Gender | 1.60 | 0.48 | 1.59 | 0.11 | 0.89 | 2.89 |
| Hypertension | 2.97 | 3.05 | 1.06 | 0.29 | 0.40 | 22.3 |

Table S2. Difference in the time to CKD3 in non-PKD2 compared with PKD2 (HR 1.54; 0.73-3.29, *P*=0.26). Male gender (HR 1.6; 0.89-2.89, *P*=0.11) and a diagnosis of hypertension (HR 2.97; 0.4-22.3, *P*=0.29) were associated with a greater risk of developing CKD3, although this did not reach significance.
